# Conservative Management of Sever’s Disease (Calcaneal Apophysitis): A Comprehensive Review of Treatment Efficacy

**DOI:** 10.7759/cureus.88779

**Published:** 2025-07-25

**Authors:** Tonyclinton C Nweke

**Affiliations:** 1 Podiatric Surgery, Wyckoff Heights Medical Center, New York, USA

**Keywords:** adolescent athletes, calcaneal apophysitis, conservative treatment, custom orthoses, heel cups, heel pain, pain reduction, pediatric sports injury, physical therapy, sever’s disease

## Abstract

Sever’s disease (calcaneal apophysitis) is a common cause of heel pain in physically active children. It results from repetitive stress on the calcaneal growth plate during adolescence. This literature review synthesizes evidence from 17 different peer-reviewed studies from PubMed using the terms “Sever’s disease” and “calcaneal apophysitis.” Approximately 243 articles were retrieved, and 17 met the inclusion criteria. The aim is to evaluate the efficacy of conservative treatments for Sever’s disease. Key interventions include the use of custom-made foot orthoses, physical therapy (including heel cord stretching and dorsiflexion strengthening), non-steroidal anti-inflammatory drugs (NSAIDs), cryotherapy, heel lifts, kinesio taping, and extracorporeal shockwave therapy (ESWT). Custom foot orthotics improved biomechanical alignment, outperforming off-the-shelf heel lifts. Physical therapy facilitated return to sport within two months. Kinesio taping enhanced function but showed comparable pain relief to placebo. Heel lifts provided short-term benefits, and ESWT showed promise but lacked robust evidence. NSAIDs and cryotherapy were effective for acute symptom management.

A tiered treatment framework was developed, prioritizing custom orthoses and physical therapy (heel cord stretching and dorsiflexion strengthening) (Tier 1), followed by adjunctive therapies like heel lifts and kinesio taping (Tier 2), emerging treatments like ESWT (Tier 3), and non-recommended options such as off-the-shelf orthoses (Tier 4). Despite methodological limitations, such as small sample sizes and heterogeneous study designs, conservative treatments consistently alleviated pain and restored function in patients with Sever’s disease. A proposed multicenter randomized controlled trial is recommended to compare custom orthoses, physical therapy, and ESWT over 12 months to standardize outcomes and clarify optimal treatment protocols.

## Introduction and background

Sever’s disease, also known as calcaneal apophysitis, is the inflammation of the calcaneal apophysis, the secondary ossification center of the calcaneus. This inflammation is accompanied by pain due to repetitive mechanical stress from the Achilles tendon (triceps surae) during growth. In this condition, the apophysis is still ossifying during adolescence, and pain and inflammation are noted at the calcaneal cartilaginous growth plate (physis). Repetitive microtrauma at the growth plate, due to disproportionate tension from the triceps surae, is a major cause of this disease. Sever’s disease is the leading cause of heel pain in children and adolescents, especially in those who are physically active, with an incidence of 3.7 per 1,000 patients. The incidence of this disease is seen in boys between the ages of 8-15 and girls between the ages of 7-12 [1]. Children with high BMI, rapid growth spurts, tight calf muscles, pes planus (flat feet), pes cavus (high-arched feet), gastrocnemius equinus (tight Achilles tendon), and the use of unsupportive footwear can all be risk factors for this disease.

A proper physical history, including the onset, duration, and exacerbating factors of pain, is required to diagnose Sever’s disease. Physical and clinical signs include tightness of the heel cord, which can be detected with the use of a Silfverskiöld test [2]. Pain is reproduced during a one-leg heel rise test. Pain and tenderness at the medial and lateral sides of the calcaneus may be elicited during a squeeze test [3], as well as the presence of aching, burning, or sharp pain at the insertion of the Achilles tendon. Sever’s disease can often be diagnosed without the use of imaging; however, imaging may still be used, particularly when symptoms are unilateral. Modalities such as X-ray can show fragmentation and help rule out fractures or neoplasms. MRI may reveal bone marrow edema from microfracture due to repeated stress, as well as soft tissue inflammation. CT scan and MRI can also help rule out stress fracture, osteomyelitis, and tarsal coalition [4].

To safely diagnose this disease, it is important to understand the differential diagnoses. These include Achilles tendinopathy, plantar fasciitis, bone cysts, tumors in the calcaneus such as osteoid osteoma, Achilles tendonitis, retrocalcaneal bursitis, tarsal coalition, posterior ankle impingement, calcaneal stress fracture, and systemic conditions like juvenile idiopathic arthritis or infectious osteomyelitis. There are several treatments available for Sever’s disease. These include rest, activity modification, the use of heel lifts, custom or prefabricated orthotics, proper footwear, physical therapy, non-steroidal anti-inflammatory drugs (NSAIDs), cryotherapy, calf stretching and strengthening, and weight loss in cases of high BMI. Education of parents, guardians, coaches, and young athletes is critical to ensure early recognition and proper intervention.

Methods

This literature review synthesizes evidence from 17 peer-reviewed studies sourced from PubMed. The search terms “Sever’s disease” and “calcaneal apophysitis” were used, retrieving approximately 243 unique articles. Relevant articles were then screened to identify studies meeting the inclusion criteria. Seventeen of those studies met the inclusion criteria. Studies were selected if they evaluated interventions, some of which include custom orthoses, physical therapy, heel lifts, kinesio taping, NSAIDs, cryotherapy, or extracorporeal shockwave therapy (ESWT), for calcaneal apophysitis. A narrative synthesis was performed due to heterogeneous study designs, and a tiered treatment framework was developed: prioritizing custom-made foot orthoses and physical therapy (Tier 1), followed by heel lifts, kinesio taping, and supportive footwear (Tier 2), ESWT (Tier 3), and non-recommended options like off-the-shelf orthoses (Tier 4).

## Review

Discussion

In 2023, a systematic review evaluated the effectiveness of conservative treatments for Sever’s disease (calcaneal apophysitis). Multiple databases were searched, including PubMed, Web of Science, Scopus, SportDiscus, and PEDro, using terminologies such as Sever’s disease, calcaneal apophysitis, and adolescent. Out of the 411 articles found, 120 were screened, and 8 randomized controlled trials were included. These trials focused on treatments for Sever’s disease such as insoles, therapeutic exercises, Kinesio taping, and foot orthoses. A PEDro scoring system was used, and an average PEDro score of 6.75 (range 4-11) was obtained, indicating good methodological quality. A bias assessment was also performed, showing four studies with low risk, three with high risk, and one with some concerns. The final results of the review showed that conservative treatments, including insoles, physical therapy, and Kinesio taping, effectively reduced pain and improved function in children with Sever’s disease, with a higher prevalence in boys. The findings emphasize the use of conservative approaches to managing Sever’s disease. However, further high-quality studies are still needed to strengthen the conclusions of this study [5].

Sweeney EA et al. conducted a randomized clinical trial comparing two braces for Sever’s disease: the Tuli’s Cheetah heel cup and Tuli’s The X Brace. Both braces were used to treat Sever’s disease (calcaneal apophysitis) in 43 barefoot athletes aged 7-14 years, mostly gymnasts. Of these athletes, 29 were girls and 3 were boys. All completed a 3-month study. Although the study began with 43 participants, 11 were lost to follow-up, leaving 32 participants to complete the study. These 32 were divided into two groups. The primary outcome was the OxAFQ-C physical score at 3 months. Secondary outcomes included OxAFQ-C school/play and emotional scores, as well as VAS pain scores at rest, during activities of daily living (ADLs), and during sports. Both groups wore their assigned braces during barefoot sports (82% for Cheetah, 64% for X Brace, p = .08) and followed standard treatments such as stretching and NSAIDs. The study found no significant differences between groups in OxAFQ-C physical (p = .80), school/play (p = .58), or emotional (p = .85) scores at 3 months, nor in VAS pain scores. However, the Cheetah group showed greater improvement in the OxAFQ-C emotional domain (p = .03). Both groups showed significant improvement in all OxAFQ-C domains and VAS scores for ADLs and sports at 2 and 3 months compared to baseline (p < .01). No adverse events were reported. The study concluded that both braces, when used alongside standard treatments, were effective in improving pain and function in barefoot athletes with Sever’s disease. There were no notable differences between the two braces [6].

RFs for Sever’s disease were investigated in a 2021 systematic review by Nieto-Gil P et al. They examined the intrinsic and extrinsic RFs, associated factors (AFs), as well as the consequences of calcaneal apophysitis (Sever’s disease) in children under 18 years old. The review was conducted using databases like PubMed and Embase up to April 2021. A total of 736 studies were identified, with 11 observational studies (2 cohort, 4 case-control, 5 cross-sectional) involving 1,265 participants (mean age: 10.72 years; 22.25% female) meeting the inclusion criteria. Most cases assessed in the study were unilateral. Intrinsic factors included several variables, with limited ankle dorsiflexion being the most common. Other factors were peak plantar pressures, foot malalignment, BMI, age, gender, and other osteochondroses. The extrinsic factors included the frequency and intensity of sports activity, although findings were conflicting. Some studies linked higher activity to increased risk, while others found no effect or even suggested that low activity increased risk. Additional factors included midfoot stiffness, mobility, and ground reaction forces. The risk of bias was moderate to low, assessed using the Newcastle-Ottawa Scale. The conclusion highlighted that limited ankle dorsiflexion, high plantar pressures, and foot malalignment are key factors associated with Sever’s disease. However, due to inconsistencies across studies, the review emphasized the need for higher-quality research with better study designs and larger sample sizes to clarify RFs, AFs, and consequences [1].

A systematic review by Fares MY et al. examined the clinical and diagnostic characteristics of calcaneal apophysitis (Sever’s disease). The study searched PubMed/Medline for English-language articles up to June 1, 2021. They identified 28 studies with 1,362 pediatric cases. All patients reported heel pain (100%). Of these, 71.4% (973 cases) were boys and 22.6% (308 cases) were girls, with a mean age of 10.69 years (10.75 for boys, ~10 for girls). The condition was bilateral in 43.2% (589 cases) and unilateral in 31.8% (433 cases). Radiography, ultrasound, and MRI were used in 26.3% of diagnoses (358 cases). Associated conditions included Osgood-Schlatter disease (1.1%) and rare cases of retrocalcaneal bursitis (0.4%). All treatments used were conservative, with 42% (573 cases) utilizing physical therapy and rest, 28% (381 cases) using orthotics, heel cups, or specialized footwear, and 0.8% (11 cases) using taping. However, 56.2% (757 cases) did not specify treatment. Outcomes were favorable: 53.7% (733 cases) showed improvement, 2.3% (32 cases) showed no improvement, and 44% did not report prognosis. The evidence suggests that Sever’s disease, commonly found in active children, is typically diagnosed clinically, though imaging can aid when needed. Conservative treatments like physical therapy, orthoses, and taping are effective, with most patients experiencing symptom relief. Educating parents and coaches on symptoms, causes, and treatments can lead to earlier diagnosis and better outcomes [7].

Kinesiotherapy’s effectiveness was assessed in male athletes with Sever’s disease. A study compared the efficacy of kinesio taping to sham taping in treating calcaneal apophysitis. Twenty-two male junior football athletes aged 10-16 years (mean age: 13.18; mean BMI: 19.6) were randomly assigned to two groups. One group received kinesio taping, and the other received sham taping. Pain (measured via VAS and foot function (measured via American Orthopaedic Foot and Ankle Society, AOFAS score) were assessed before treatment and at 1 week, 1 month, 3 months, and 6 months post-treatment. Both groups showed significant pain reduction at all follow-up points compared to baseline (p < 0.05), with no significant difference in VAS scores between the groups (p > 0.05). However, the kinesio taping group had significantly higher AOFAS scores at 1 and 3 months compared to the sham group (p < 0.05), indicating better functional improvement. Post-treatment, the kinesio group’s AOFAS score was 99.7 ± 0.9, and the sham group’s was 97.4 ± 3.9. VAS scores were 0.1 ± 0.3 and 0.4 ± 0.5, respectively (p > 0.05). The outcomes demonstrate that kinesio taping is effective for improving ankle and foot function in athletes with Sever’s disease, although its effect on pain reduction is comparable to a placebo. Due to its lack of serious side effects, kinesio taping can be used alongside or as an alternative to other treatments like massage or manual therapy to relieve plantar fascia tension [8].

German youth soccer players were analyzed for calcaneal apophysitis (Sever’s disease) incidence and RTP time at a youth soccer academy (2009-2018). Among 612 male adolescent athletes, 4,326 total injury cases were recorded. The study identified 22 cases of calcaneal apophysitis in 19 athletes, representing 0.51% of all musculoskeletal complaints. The incidence rate was 0.36 per 100 athletes per year. The mean age at diagnosis was 11.8 ± 2.1 years. The condition was unilateral in 20 cases and bilateral in 2 (10.5%). Three athletes (15.8%) experienced recurrent injuries. Flatfoot and hyperpronation were noted in 26.3% of cases, while 73.7% had normal foot biomechanics. The mean RTP time was 60.7 days (95% CI: 32.0-89.5), with 77.3% of cases classified as severe (RTP > 4 weeks) and 22.7% as moderate. Bilateral cases had significantly longer RTP (209.5 days) compared to unilateral cases (45.9 days, p = 0.017). Recurrent cases also had extended RTP (181.0 days) compared to primary diagnoses (41.7 days, p = 0.002). No correlation was found between RTP and age (p = 0.97) or BMI (p = 0.54). Most cases occurred early in the season or after winter break, suggesting a link to increased activity following rest periods. This study is the first to quantify the incidence of calcaneal apophysitis in elite youth soccer players, finding it comparable to the general population (0.36 vs. 0.37 per 100 per year) but lower than in pediatric clinics due to selection bias. The findings highlight that bilateral or recurrent cases are RFs for prolonged RTP. Conservative treatments, including cooling, NSAIDs, stretching, and orthotics (heel lifts, cups, or pads), were typically used, with immobilization in severe cases. The study underscores the need for further prospective research to better understand etiology and develop preventive strategies [9].

Health-related QOL from both child and parent perspectives was examined in children with Sever’s disease in an Australian trial. 133 children were recruited, and 124 participated. Nine patients were excluded for reasons such as resolved pain or suspected other conditions, and 101 completed the OxAFQ-C at baseline, 1, 2, 6, and 12 months. The OxAFQ-C assessed physical, school, emotional, and footwear domains. The agreement between child and parent responses varied. The physical domain agreement ranged from poor (0.06) to good (0.77), footwear from poor (0.09) to good (0.66), and school and emotional domains from moderate (0.46) to good (0.77). Agreement improved over time in physical, emotional, and school domains, but footwear agreement fluctuated. Parents initially reported a greater QOL impact than children, though perceptions converged over the 12-month follow-up. Given its substantial impact on QOL, calcaneal apophysitis requires treatment approaches that reflect both child and parent perspectives. Further research comparing OxAFQ-C scores with other lower limb conditions is needed to assess the condition’s relative impact [10].

A Level 1 therapeutic randomized, single-blind clinical trial by Wiegerinck JI et al. compared three different conservative treatments for calcaneal apophysitis (Sever’s disease) in children aged 8-15 with heel pain for at least 4 weeks and a minimum Faces Pain Scale-Revised score of 3. The treatments were: a wait-and-see approach, heel raise inlays (ViscoHeel), and supervised eccentric exercise physical therapy. Each of these treatments lasted 10 weeks. From October 2010 to September 2013, 117 patients were screened, 101 were randomized, and 98 completed the study. Three patients dropped out due to symptom resolution. The study population was 75% male, with a mean age of 10.6 years. Outcomes measured at baseline, 6 weeks, and 3 months included pain (Faces Pain Scale-Revised), function (OAFQ, for children and parents), and patient satisfaction. All three treatments significantly reduced heel pain and improved function at all follow-up points (p < 0.005). At 6 weeks, the heel raise group reported higher satisfaction (p = 0.01 vs. physical therapy, p = 0.001 vs. wait-and-see). The heel raise group also reported better OAFQ scores for children compared to wait-and-see (p = 0.009). However, the physical therapy group showed better OAFQ parent scores than the wait-and-see group (p = 0.004). By 3 months, no significant differences were found between groups in any outcome. All three interventions, wait-and-see, heel raise inlays, and physical therapy, are equally effective in reducing heel pain and improving function in calcaneal apophysitis, suggesting clinicians should discuss treatment preferences with patients and parents, as all options yield similar results [11].

Custom orthoses versus heel lifts were evaluated in a 2019-2020 randomized clinical trial for Sever’s disease by Alfaro-Santafé J et al. They compared the effectiveness of custom-made polypropylene foot orthoses versus off-the-shelf heel lifts for relieving pain in children aged 9-12 with calcaneal apophysitis (Sever’s disease). This study was conducted over 12 weeks. Treatment A was custom-made polypropylene foot orthoses, while Treatment B was off-the-shelf heel lifts. From June 2019 to February 2020, 234 children were screened, and 208 were included, with 104 assigned to each group. Baseline characteristics, including BMI, Foot Posture Index (FPI-6), and the lunge test, showed no significant differences between groups, indicating normal weight, shortened triceps surae muscles, and pronated feet. Algometry (pressure pain threshold in kgf) and the VAS were used to assess pain. Only four children were lost to follow-up in Treatment A and five in Treatment B. Compliance was high throughout the study. Both groups showed significant pain reduction (p < 0.001); however, the custom-made orthoses group had greater improvements: algometry increased by 2.0 ± 0.5 kgf (53.4%, 95% CI: 47.1-59.7) compared to 0.6 ± 0.6 kgf in the heel lift group, and VAS decreased by 68.5 ± 15.4 points (68.6%, 95% CI: 62.7-74.5) versus 14 ± 17.7 points in the heel lift group (p < 0.001 for inter-group differences). The evidence indicates that custom-made foot orthoses are more effective than heel lifts for reducing pain in calcaneal apophysitis, suggesting their use as a preferred clinical treatment. It is important to note that future research should compare orthoses with other treatments like exercises, physiotherapy, or medications to further evaluate pain relief strategies [12].

Another study investigated the usefulness of combining custom-made insoles with a home exercise program on foot pressure distribution, gait parameters, and pain. This study included 40 children aged 8-15 with calcaneal apophysitis (Sever’s disease). The one-group pretest-posttest study included 34 participants who completed a 4-week intervention (3 days/week). Four were excluded for not meeting the criteria, while two were lost to follow-up. Of the participants, 67.65% were male and 32.35% were female. Nineteen participants had right foot involvement, and 15 had left foot involvement. Pain was assessed using the VAS, and gait parameters were measured using the Zebris FDM-THM-S treadmill system before and after treatment. Post-treatment results showed significant improvements (p < 0.05) in stride length (mean difference: 4.26), stance phase percentage, swing phase percentage, and gait speed. However, there were no significant changes in step width or cadence (p > 0.05). Foot pressure distribution shifted significantly, with decreased forefoot and rearfoot pressure, increased midfoot pressure (p < 0.05), and effect sizes ranging from 1.08 to 1.22. Pain during activity decreased significantly (mean difference: 4.08, Cohen’s d = 4.58, p < 0.05). The outcomes confirm that the combination of custom-made insoles and exercise effectively reduces pain and rearfoot pressure, improves gait parameters, and prevents adaptive walking impairments in children with calcaneal apophysitis [13].

In a 12-month factorial randomized trial, James AM et al. evaluated the benefit of footwear and foot orthoses for treating calcaneal apophysitis (Sever’s disease). The study included 124 children aged 8-14 years, recruited from podiatry caseloads at Monash Health and Peninsula Health. A 2×2 factorial design compared two factors: in-shoe orthoses (heel raises vs. prefabricated orthoses) and footwear replacement (new footwear vs. no replacement). The primary outcome was functional disability, measured by the OxAFQ, with secondary outcomes including pain and ankle dorsiflexion range. Assessments were conducted at baseline, 1, 2, 6, and 12 months. At 1 and 2 months, heel raises showed significant improvement in the physical domain of the OxAFQ compared to prefabricated orthoses (p = 0.04). However, at 6 and 12 months, there were no significant main or interaction effects for any outcome measure across the treatment groups. Heel raises offered a relative advantage over prefabricated orthoses for reducing functional disability in the short term (up to 2 months). Beyond 2 months, no treatment option was superior. This suggests that treatment choices may depend on clinical judgment, cost, or patient preference in persistent cases [14].

Factors associated with Sever’s disease were explored in a case-control study conducted in a soccer academy with 106 athletic boys. The study compared 53 boys with Sever’s disease to 53 age-matched healthy controls. Assessments included BMI, dynamic plantar pressures, plantar surface contact area, center of pressure (COP) velocity, and ankle dorsiflexion range of motion (using goniometry to identify gastrocnemius equinus and gastrocnemius-soleus equinus) using a digital pressure sensor platform. Boys with Sever’s disease had significantly higher BMI and higher average and maximum peak plantar pressures at the heel (Cohen’s d > 3, p < 0.001) during 20% and 35% of the stance phase. They also had moderately higher forefoot pressures at 92% of the stance phase (p < 0.001). Additionally, they exhibited significantly slower COP velocity at 20% (27.74 vs. 55.64 mm/s), 35% (34.99 vs. 66.21 mm/s), and 92% (34.59 vs. 31.48 mm/s) of the stance phase (p < 0.01). Plantar surface contact areas were similar between both groups. Boys with Sever’s disease were observed to be eight times more likely to have bilateral gastrocnemius equinus than the healthy control group. High heel plantar pressures and low COP velocity are associated with Sever’s disease; however, it is unclear whether these are causes or effects. Gastrocnemius equinus may be a predisposing factor. Further research is needed to clarify causality and inform prevention strategies [15].

The effectiveness and short-term safety of focused ESWT for apophyseal injuries were analyzed in a retrospective study involving 22 growing athletes. This included 15 athletes with Osgood-Schlatter disease and 7 athletes with calcaneal apophysitis (Sever’s disease), treated at a university hospital’s sports medicine unit. Low-energy ESWT (≤ 0.1 mJ/mm²) was applied using electrohydraulic technology and a clinical focusing technique. Patients received 1-3 sessions. Pain relief ranged from 50-100% (median: 100%) after the first session, with 14 patients (63.3%) achieving complete relief after one session, 7 (31.8%) after two sessions (within 1-2 weeks), and 1 after three sessions. RTP time ranged from 2-11 weeks (median: 2 weeks), with 14 patients (63.3%) returning in 2 weeks, 7 (31.8%) in 4 weeks, and 1 (4.5%) in 11 weeks. No adverse events or recurrences were reported within 3 months post-treatment, and no skeletal deformities were observed. ESWT appears to be a safe, effective, non-invasive treatment that may shorten recovery and enable early return to sports for youth with apophyseal injuries. However, a prospective controlled trial with long-term follow-up (at least 1 year) is needed to confirm long-term efficacy and safety, particularly regarding effects on growing epiphyses [16].

Interventions for reducing pain and maintaining physical activity in children and adolescents aged 8-15 with calcaneal apophysitis were reviewed in a 2012 systematic analysis by James AM et al. Nine databases were searched up to May 2012, yielding nine studies. The included studies were: three randomized clinical trials (Level 2), one cohort study (Level 3), and four case series (Level 4). The trials compared heel raises to orthoses or orthoses to no treatment, while other studies used multiple concurrent treatments. Methodological quality was assessed using the PEDro scale, which revealed generally low scores due to study design issues. Treatments in the study were categorized into two groups: the first was inflammation and pain reduction strategies (e.g., rest, ice, pharmaceuticals); the second was mechanical strategies (e.g., heel raises, orthoses, taping, stretching). Limited evidence suggested that orthoses provided greater short-term pain relief than heel raises. Meta-analysis was not possible due to inadequate data reporting and varied interventions. While heel raises and orthoses may offer pain relief, the evidence is weak due to methodological flaws and small sample sizes. Primary care practitioners should interpret these findings cautiously. Further high-quality randomized controlled trials with validated pain and function measures and larger sample sizes are needed to establish effective treatment approaches for calcaneal apophysitis [17].

Conservative treatments were evaluated in 85 children with Sever’s disease. Bilateral heel involvement occurred in 61% (52 patients). Pronation was the most frequently associated foot condition, noted in 16 patients. It was observed that pain was exacerbated by specific sports, especially soccer, in 68 patients. All patients underwent a conservative treatment regimen that included physical therapy and lower extremity stretching focused on heel cords and ankle dorsiflexion strengthening. Soft Plastizote orthotics or heel cups were used in 98% of cases, and proper athletic footwear was recommended. All patients improved, returning to their chosen sport within an average of 2 months (range: 1-6 months), with boys averaging 2.2 months and girls 1.6 months. Two boys experienced recurrences one year after initial symptoms (one with bilateral involvement and the other unilateral); however, both were successfully treated with the same regimen and resumed unlimited activity. Conservative management, including physical therapy, orthotics, and proper footwear, proved beneficial for managing calcaneal apophysitis, with a low recurrence rate [18].

A systematic review study by Bourke J et al. assessed the efficacy and safety of heel lifts for lower limb musculoskeletal conditions, including calcaneal apophysitis (Sever’s disease), mid-portion Achilles tendinopathy, and plantar heel pain. The review searched Ovid MEDLINE, AMED, EMCARE, CINAHL Plus, and SPORTDiscus up to May 2024 and identified eight eligible trials (n = 903) with randomized, quasi-randomized, or non-randomized designs comparing heel lifts to interventions such as exercise, ultrasound, cryotherapy, orthotics, stretching, footwear, activity modification, felt pads, or indomethacin. Outcomes included pain, disability/function, participation, QOL, and adverse events, evaluated at 12 weeks or the closest time point. The risk of bias and evidence certainty were assessed using the GRADE approach. Clinically significant differences were shown in only 2 out of the 47 outcomes. Low-certainty evidence from one trial (n = 199) indicated custom orthotics reduced pain more than heel lifts for calcaneal apophysitis at 12 weeks (55.7 points on a 100 mm VAS, 95% CI: 50.3-61.1). Very low-certainty evidence from one trial (n = 62) suggested heel lifts improved pain and function more than indomethacin for plantar heel pain at 12 months (35.5 points on Foot Function Index, 95% CI: 21.1-49.9). There were no clinically meaningful differences in other comparisons, including heel lifts versus exercise or prefabricated orthotics. Most evidence was of very low to low certainty, indicating uncertainty in results. Given the limited evidence for the efficacy of heel lifts, rigorous trials are needed to clarify their clinical role in these conditions [19].

A novel technique for creating custom-made foot orthoses using vacuum forming on the non-load-bearing foot to treat calcaneal apophysitis in male children was studied. The orthoses were molded directly to the foot using a combination of materials. These materials were polyvinyl chloride and polyester resins, 30 Shore A hardness polyethylene-ethylene-vinyl-alcohol (148 kg/m³ density), and a 22 Shore A polyurethane cushioning heel cup. The study aimed to describe this off-loading technique as well as assess its suitability for managing Sever’s disease pain. The VAS was used to assess pain at three time points: baseline, M1 (first follow-up), and M2 (second follow-up). Significant reductions in pain were observed across all comparisons (baseline vs. M1, M1 vs. M2, baseline vs. M2), with large effect sizes, particularly between baseline and M1. This direct-molding and non-load-bearing technique offers a promising alternative to traditional insoles for managing Sever’s disease pain, potentially improving individual adaptation. This approach is clinically relevant; however, further research is needed to validate its broader application [20].

Analysis and proposed tiered treatment framework

The reviewed studies consistently demonstrate that conservative treatments effectively manage Sever’s disease, a leading cause of heel pain in patients, with significant pain relief and functional improvements. Custom-made foot orthoses stand out as a primary treatment for Sever’s disease, with evidence showing a 68.6% reduction in pain (VAS) and a 53.4% increase in pressure pain threshold over 12 weeks, significantly surpassing off-the-shelf heel lifts [12]. Novel designs, such as vacuum-formed orthoses, have also shown substantial benefits, emphasizing the importance of individualized adaptation [20]. Combining custom insoles with a home exercise program reduced pain (VAS mean difference 4.08) and enhanced gait parameters, underscoring biomechanical benefits [13]. Physical therapy, particularly heel cord stretching and dorsiflexion strengthening, also proves highly effective, as Micheli LJ and Ireland ML noted, with return to sport within an average of 2 months when paired with orthotics [18]. Comparable pain reduction and functional improvement across eccentric exercise, heel raises, and a wait-and-see approach at 3 months have been reported, suggesting flexibility in treatment options [11]. Furthermore, therapeutic exercises, insoles, and kinesio taping have been shown to improve function and reduce pain [5].

Kuyucu E et al. found that kinesio taping improved foot function (AOFAS scores) more than sham taping at 1 and 3 months, although pain relief was comparable, indicating its role as a supportive adjunct [8]. Heel lifts and cups, evaluated by Sweeney EA et al., provided significant pain relief and functional gains in barefoot athletes, with no notable differences between Tuli’s Cheetah heel cup and Tuli’s The X Brace [6]. However, James AM et al. reported that heel raises offered only short-term (1-2 months) advantages over prefabricated orthoses, and Bourke J et al. confirmed that custom orthotics outperformed heel lifts at 12-week follow-ups [14, 19]. ESWT achieved 50-100% pain relief with a median 2-week RTP time in seven athletes, but the small sample size limits its generalizability [16]. Rest, used in 42% of cases per Fares MY et al. alongside NSAIDs and cryotherapy, remains foundational for acute symptom management, with 53.7% of patients showing improvement [7]. Meanwhile, the use of cooling, NSAIDs, and orthotics, though effective, was associated with extended RTP times in severe cases [9]. Proper footwear, recommended by Micheli LJ and Ireland ML supports other interventions but lacks standalone efficacy, as James AM et al. found no long-term benefit from footwear replacement [14, 18]. A wait-and-see approach, while effective in mild cases, risks prolonged recovery in active children [11].

Based on all these findings, a tiered treatment approach is proposed. Tier 1 categories represent first-line treatments with the strongest evidence for significant pain relief and functional improvement, making them the preferred initial interventions for Sever’s disease. This tier includes custom-made foot orthoses, which studies like Alfaro-Santafé J et al. [12] and Gijon-Nogueron G et al. [20] showed reduced pain by up to 68.6% and improved biomechanical alignment, and physical therapy (heel cord stretching and dorsiflexion strengthening), supported by Micheli LJ and Ireland ML [18] and Wiegerinck JI et al. [11] for enabling return to sport within 2 months and enhancing function. NSAIDs and cryotherapy are also included, as Hernandez-Lucas P et al. [5] and Sweeney EA et al. [6] demonstrated their effectiveness in managing acute inflammation and pain.

Tier 2 encompasses adjunctive treatments that are moderately effective and serve as supportive or alternative options when Tier 1 treatments are insufficient or unavailable. Tier 2 comprises heel lifts and heel cups, which Sweeney EA et al. [6] and James AM et al. [14] found provide short-term pain relief; kinesio taping, which Kuyucu E et al. [8] showed improved function; and supportive footwear, recommended by Micheli LJ and Ireland ML [18] to complement other therapies.

Tier 3 includes emerging treatments with limited but promising evidence, such as ESWT, which Shafshak T and Amer MA [16] reported achieved rapid pain relief but lacks robust validation due to small sample sizes. These are considered for refractory cases after Tier 1 and 2 options are exhausted.

Finally, Tier 4 lists treatments not recommended due to insufficient evidence or inferior outcomes, such as off-the-shelf orthoses, which Alfaro-Santafé J et al. [12] and James AM et al. [14] found less effective than custom orthoses, and non-standard medications like indomethacin, which Bourke J et al. [19] showed offer no significant benefit over NSAIDs. This tiered framework will help podiatrists (foot and ankle surgeons) and other physicians to prioritize treatments based on evidence strength, ensuring efficient management of Sever’s disease while minimizing risks and costs, with the flexibility to tailor interventions to patient needs and response.

## Conclusions

This literature review confirms that conservative treatments are effective in managing Sever’s disease, providing both heel pain relief and functional restoration in affected patients. These interventions alleviate symptoms and restore mobility in active children. Custom-made foot orthoses and physical therapy, particularly heel cord stretching and dorsiflexion strengthening, emerged as first-line (Tier 1) treatments, demonstrating significant pain reduction (up to 68.6% on the VAS) and functional improvement, with return to sport achievable within two months. Adjunctive therapies (Tier 2), including heel lifts, kinesio taping, and supportive footwear, offered moderate benefits. ESWT, classified as Tier 3, shows promise but requires further validation and supporting evidence. Tier 4 treatments, such as off-the-shelf orthoses and non-standard medications like indomethacin, are not recommended due to inferior outcomes. NSAIDs and cryotherapy remain foundational for acute symptom relief. Despite these encouraging findings, limitations such as small sample sizes, low-certainty evidence, and inconsistent long-term outcome reporting underscore the need for high-quality, standardized research. A proposed multicenter randomized controlled trial with a large cohort (n = 300) over 12 months could address these gaps, provide robust evidence to optimize treatment protocols, further validate the tiered treatment framework, and ultimately improve clinical outcomes for Sever’s disease.
